# Minding the Gap: Clinical Manifestations of a Rare Type IV Hiatal Hernia

**DOI:** 10.7759/cureus.9275

**Published:** 2020-07-19

**Authors:** Shaunak Patel, Silpa Yarra, Shayan Owji, Jacob E Benavidez, Quan D Nguyen

**Affiliations:** 1 Radiology, University of Texas Medical Branch, Galveston, USA

**Keywords:** hiatal hernia

## Abstract

Hiatal hernias are classified according to the increasing severity of protruding intra-abdominal viscera through the esophageal hiatus (types I-IV). Herein is the case of an elderly patient presenting with recent-onset dyspnea, postprandial gastroesophageal reflux, and hypoxemia. Imaging revealed a rare type IV hiatal hernia implicating the stomach and part of the pancreas. This case highlights the seemingly benign clinical manifestations of a massive hiatal hernia, despite its ability to complicate treatment or exacerbate comorbid conditions.

## Introduction

Hiatal hernias often result from the protrusion of the stomach into the thoracic cavity. There are four types of hiatal hernias, graded in severity I through IV [1]. Type I hiatal hernias are sliding hiatal hernias that account for the majority of all hiatal hernias and are characterized by widening of the esophageal hiatus, which allows the gastroesophageal (GE) junction and sometimes the gastric cardia to be displaced above the diaphragm. Type I hiatal hernias are most commonly associated with symptoms of gastroesophageal reflux disease (GERD). Type II hiatal hernias are paraesophageal hernias where only the gastric fundus, not the GE junction, herniates through the esophageal hiatus alongside the esophagus. Severe clinical manifestations, such as volvulus of the stomach, can occur in type II hiatal hernias, due to the fixed nature of the GE junction. Type III hiatal hernias are a mixture of type I and type II hiatal hernias. Type III hiatal hernias present with a paraesophageal herniation in addition to the herniation of the GE junction. Finally, type IV hiatal hernias are massive herniations defined by the presence of the stomach and other abdominal organs into the thoracic cavity. This occurs due to a large defect in the phrenoesophageal membrane, as well as an increased laxity in the esophageal hiatus, providing more area for organ protrusion. Type IV hiatal hernias are considered rare, accounting for less than 5% of all hiatal hernias [2]. This case presents a patient with a type IV hiatal hernia resulting in atypical symptoms ultimately caused by mass effect.

## Case presentation

**Figure 1:**
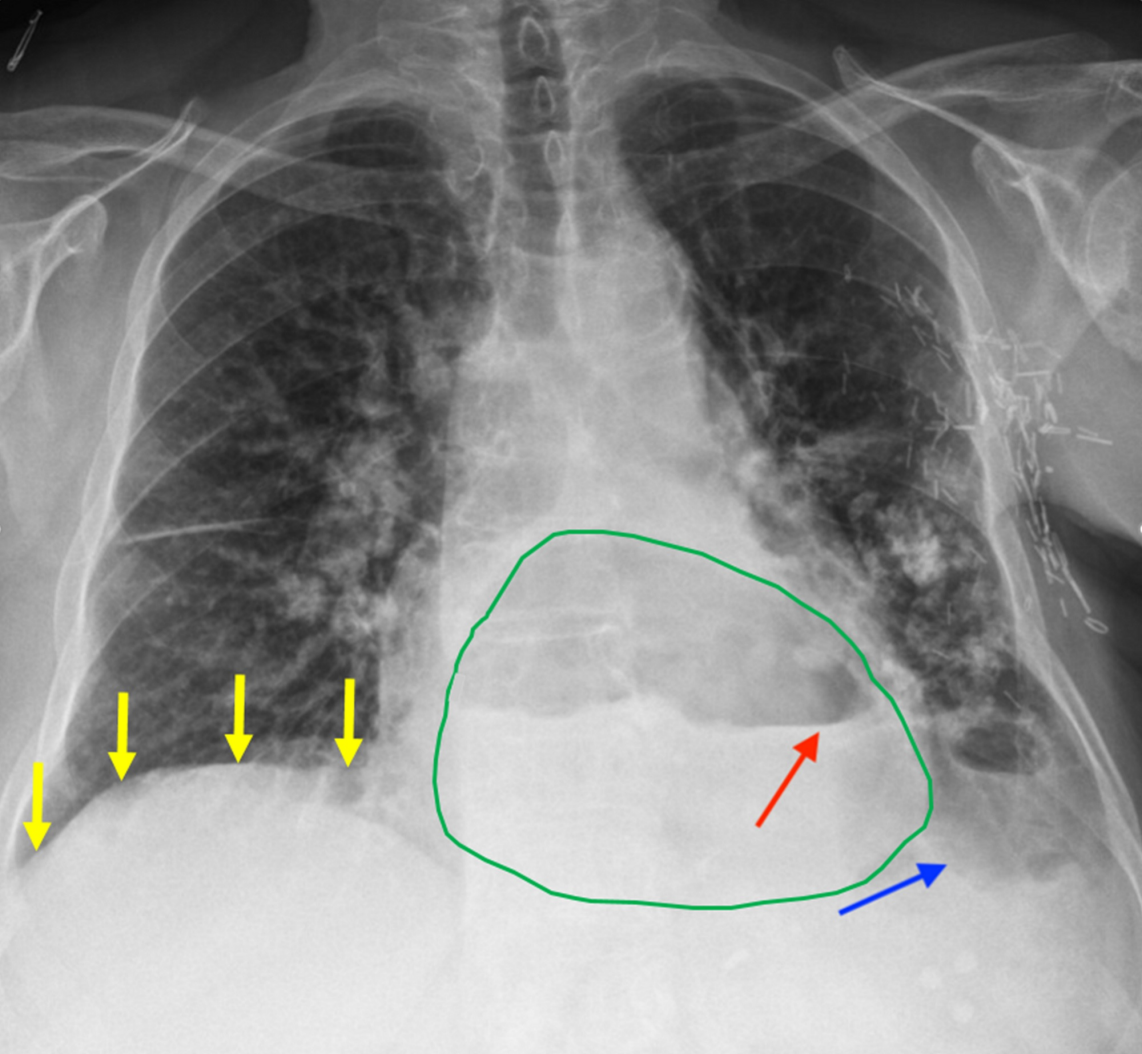
Chest radiograph revealing a massive herniation of intra-abdominal contents Abdominal contents herniating (green outline) above the level of the diaphragm (yellow arrows) into the thorax are observed. The air-fluid level of the stomach (red arrow) is identified above the level of the diaphragm. A small pleural effusion is seen in the lower lobe of the left lung (blue arrow).

A 77-year-old woman presented with a two-month history of increasing dyspnea on exertion, fatigue, and severe anasarca extending into the lower extremities. She has no other complaints except for mild symptoms associated with postprandial GE reflux, mild paroxysmal nocturnal dyspnea, and orthopnea. The patient denied any history of cardiac or respiratory issues, as well as fevers, night sweats, and unintentional weight loss. During physical examination, vital signs were stable except for an oxygen saturation of 92%, indicating hypoxia. No abnormal physical examination findings were observed in the patient other than pitting edema, moderate erythema, and warmth of the lower extremities. Laboratory studies, including a complete blood count and basic metabolic panel, were within normal limits. A subsequent chest radiograph demonstrated pulmonary edema, a small left pleural effusion, and a prominent hiatal hernia (Figure 1).

**Figure 2:**
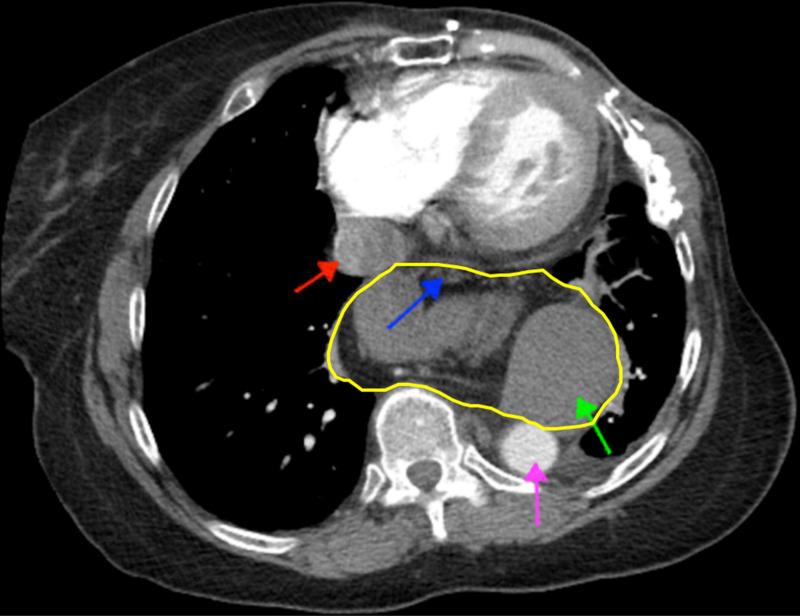
Axial CT angiogram of the chest featuring a distorted mediastinal anatomy and pleural cavity due to a massive hiatal hernia Retrosuperior displacement of the aorta (purple arrow) and inferior vena cava (red arrow) without compression of critical vasculature is appreciated, as the hiatal hernia with intra-abdominal contents (yellow outline) is seen protruding into the thorax. The intra-abdominal contents within the hiatal hernia include the pancreas and associated fat content (blue arrow) and stomach (green arrow).

**Figure 3:**
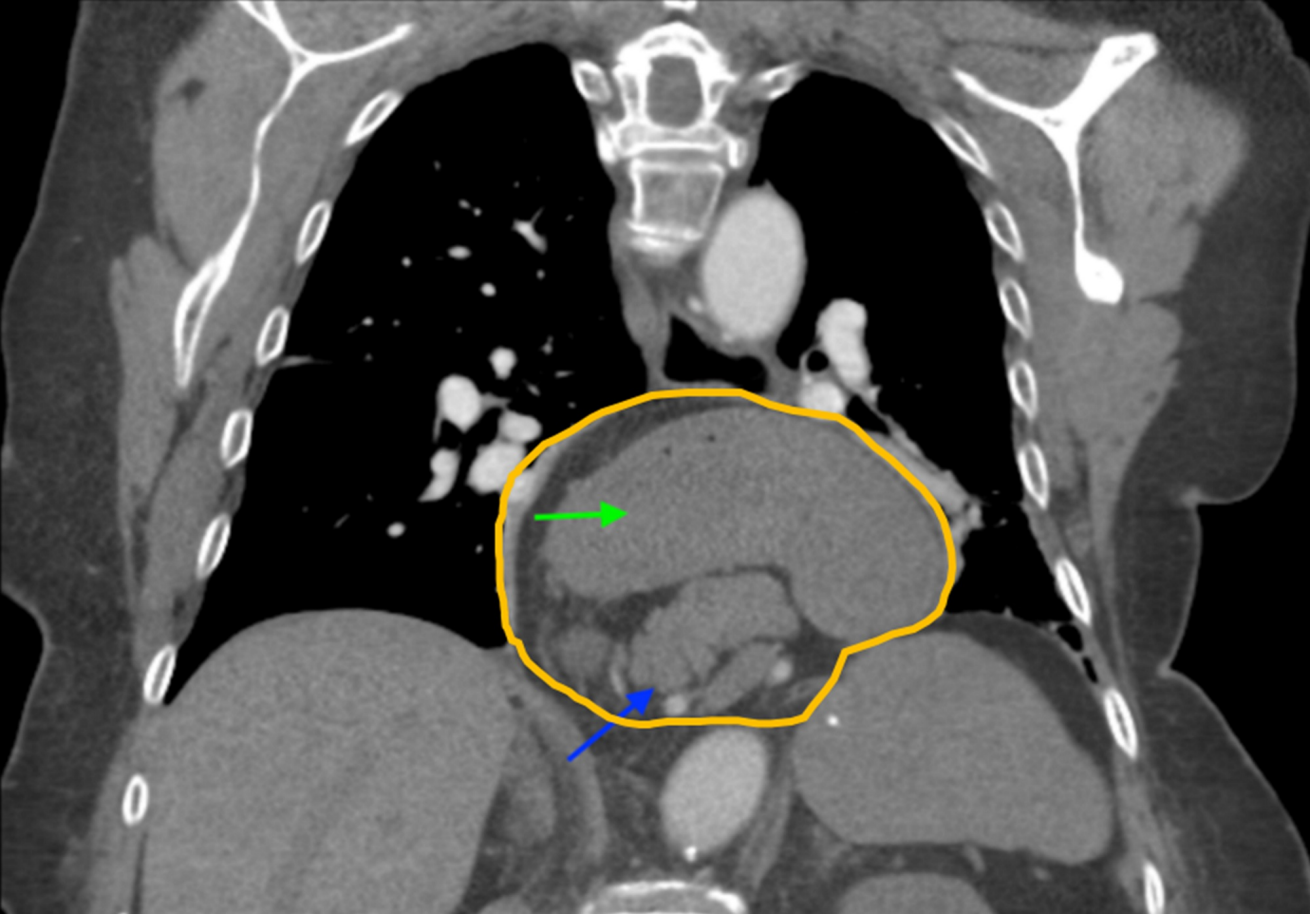
Coronal CT angiogram of the chest featuring a massive type IV hiatal hernia with intra-abdominal contents A massive type IV hiatal hernia (orange outline) containing a large portion of the pancreas (blue arrow) and stomach (green arrow) is shown entering the thorax and obscuring the view of critical vasculature.

A CT scan of the chest was performed, which demonstrated a type IV hiatal hernia involving the stomach and a portion of the pancreas, which also displaced the aorta and inferior vena cava (Figures 2, 3).

The patient was then admitted to the hospital for further work-up and appropriate treatment of hypoxia, as well as pitting and peripheral edema. The patient underwent a transthoracic echocardiography during her hospital course where a preserved ejection fraction was appreciated. Her symptoms of increased dyspnea on exertion, postprandial GE reflux, and mild hypoxia were attributed to chronic aspiration pneumonia secondary to the massive hiatal hernia. Oral diuretics were used to treat the patient’s peripheral edema. The massive hiatal hernia displacing the aorta and inferior vena cava seen on the CT scan of the chest could be a factor in her symptoms; however, it was not considered one the sentinel cause. The patient was kept on oral diuretic therapy and nasal cannula oxygen during her hospital stay, and later referred to gastroenterology and pulmonology for evaluation of her type IV hiatal hernia.

## Discussion

Hiatal hernias are the protrusion of the stomach and occasionally other intra-abdominal organs into the thoracic cavity. Type IV hiatal hernias are extremely rare and include the protrusion of the stomach and other abdominal organs through the esophageal hiatus, accounting for less than 5% of all hiatal hernias [1,2]. In fact, the combination of types II, III, and IV hiatal hernias account for about 5% of all hiatal hernias. Within that 5%, 2%-5% are solely type IV hiatal hernias [3].

The causes of hiatal hernias are widely unknown; however, they are identified as increasing in incidence with an increase in age due to the laxity of the diaphragmatic crura during the aging process and repeated occurrences of elevated intra-abdominal pressure [4,5]. The most common symptoms of type IV hiatal hernias are heartburn, regurgitation, progressive dysphagia, chest pain, and nausea/vomiting [3]. However, complications of type IV hiatal hernias can involve gastric volvulus, perforations, incarceration of organs, and, in the preceding patient’s case, increased dyspnea and recurrent aspiration pneumonia due to progressive dysphagia [4,5].

There are multiple diagnostic techniques that are of use in diagnosing hiatal hernias. Using some of these modalities can be challenging because of the irregularities seen in the anatomy of the GE junction during respiration, movement, and swallowing [6]. Barium swallow radiography provides important details of the upper gastrointestinal tract, including insight of disease pathology, in this case, the size of the herniated portion of the stomach as well as the position of the GE junction. It is well documented that barium swallow is a useful tool in the diagnosis of hiatal hernias [7]. Esophagogastroduodenoscopy (EGD) is also used for real-time evaluation of the esophageal, stomach, and duodenal mucosa [6]. However, a disadvantage of EGD, especially in the case of the preceding patient, is the inability to appreciate large hiatal hernias [8]. Additionally, CT scans are useful in visualization of hiatal hernias and provide valuable information on the type of hiatal hernia and involved organs. In the preceding case, a type IV hiatal hernia was confirmed on CT, based on the proximal displacement of the majority of the stomach along with the pancreas through the diaphragmatic hernia.

There is a particular prevalence of respiratory complications in patients with hiatal hernias [9]. This is largely due to the chronic GE reflux which is often observed in the setting of hiatal hernia. In patients with hiatal hernia and concomitant respiratory complications, GERD may be associated with episodes of bronchoaspiration, which can trigger respiratory complications ranging from mild to life-threatening. A patient with GERD secondary to hiatal hernia may present with milder pulmonary manifestations, such as temporary dyspnea, or with more serious complications, such as severe bronchoconstriction and acute respiratory failure [10]. The aspiration of gastric and esophageal contents into the lungs may lead to bronchial irritation and aspiration pneumonia [11]. Patients with hiatal hernias who developed aspiration pneumonia can present with bronchiolitis, centrilobular wall thickening, lung opacities, and ground-glass infiltrates on imaging [12]. Given the association between hiatal hernia and pulmonary complications, our patient’s diminished oxygen saturation is suggestive of a pulmonary manifestation of her extensive GE reflux, secondary to a type IV hiatal hernia.

It is important that management of hiatal hernias be carefully considered in the care of patients with concurrent cardiac complications. Patients with both an existing history of heart failure with a preserved ejection fraction and pulmonary dysfunction can quickly decline with the added stress of a massive hiatal hernia which impedes perfusion capacity. The clinical presentation of a patient with existing heart failure and acute hypoxia from a large type IV hiatal hernia is rare, but would be straightforward to identify with the use of current imaging modalities available. In the preceding case presented, constriction of the patient’s pulmonary vasculature in response to the hiatal hernia would increase hydrostatic pressure and lead to severe pitting edema. Although less common, it is also worth noting that hiatal hernias, particularly large ones such as those seen in type IV, may lead to similar cardiopulmonary complications via direct compression of mediastinal structures [13].

## Conclusions

Heightened awareness and consideration for hiatal hernias, accompanied by non-invasive diagnostic techniques, may promote timely detection and monitoring. Although rare, massive type IV hiatal hernias can cause the protrusion of abdominal contents into the thoracic cavity, leading to life-threatening complications. The reduced respiratory capacity may then cause or worsen hypoxia in certain patients, exacerbate existing heart failure, and contribute to edema along with a host of other complications.
